# Verification of the Seismic Performance of a Rigidly Connected Modular System Depending on the Shape and Size of the Ceiling Bracket

**DOI:** 10.3390/ma10030263

**Published:** 2017-03-06

**Authors:** Seungjae Lee, Jaeseong Park, Euishin Kwak, Sudeok Shon, Changhoon Kang, Hosoon Choi

**Affiliations:** 1 Interdisciplinary Program in Creative Engineering, Korea University of Technology and Education, Cheonan 31253, Korea; leeseung@koreatech.ac.kr (S.L.); jspark@koreatech.ac.kr (J.P.); euishin@koreatech.ac.kr (E.K.); 2 Department of Architectural Engineering, Korea University of Technology and Education, Cheonan 31253, Korea; 3 Structure Design Team, Hyundai Engineering Co., Ltd., 75 Yulgok-ro, Jongno-gu, Seoul 03058, Korea; judeman@hec.co.kr (C.K.); hosoon.choi@hec.co.kr (H.C.)

**Keywords:** modular system, cyclic loading test, seismic performance, column-beam connection, rigidly-connected joint, ceiling bracket

## Abstract

Modular systems have been mostly researched in relatively low-rise structures but, lately, their applications to mid- to high-rise structures began to be reviewed, and research interest in new modularization subjects has increased. The application of modular systems to mid- to high-rise structures requires the structural stability of the frame and connections that consist of units, and the evaluation of the stiffness of structures that are combined in units. However, the combination of general units causes loss of the cross-section of columns or beams, resulting in low seismic performance and hindering installation works in the field. In addition, the evaluation of a frame considering such a cross-sectional loss is not easy. Therefore, it is necessary to develop a joint that is stable and easy to install. In the study, a rigidly connected modular system was proposed as a moment-resisting frame for a unit modular system, and their joints were developed and their performances were compared. The proposed system changed the ceiling beam into a bracket type to fasten bolts. It can be merged with other seismic force-resisting systems. To verify the seismic performance of the proposed system, a cyclic loading test was conducted, and the rigidly connected joint performance and integrated behavior at the joint of modular units were investigated. From the experimental results, the maximum resisting force of the proposed connection exceeded the theoretical parameters, indicating that a rigid joint structural performance could be secured.

## 1. Introduction

### 1.1. Research Background

The potential of modular systems has been recognized in the construction market, going beyond the role of temporary on-site structures. Accordingly, various types of modular structure systems have been suggested in many developed countries, and various attempts have been made to design them, with structural stability and easy construction as the main considerations. The demand for modular-type structures has been increasing with time, and such structures are used in diverse applications, such as houses, schools, and hospitals. Module development and performance evaluation for mid- to high-rise buildings have drawn the attention of a number of construction companies of late [1,2].

The studies on modular construction have been diverse lately, including those on BIM(Building Information Modeling)-based project planning [3], structural performance in extreme environments [4] or hysteresis behavior under a seismic load [5], construction algorithms [6], and the demand for and application of modular construction methods [7,8,9], as well as case studies of modular buildings [2]. These studies have developed platforms in connection with BIM, devised methods that can achieve durability and structural stability under various conditions, and with simple assembly. They have also made efforts to demonstrate the validity of the construction algorithms developed for installation. Through this process, various ideas and techniques have been developed, and in the long run, the modularization of all the structures will increase their economic feasibility.

In the case of a modular system, various systems have been suggested to minimize the on-site processes. In particular, securing seismic performance at the joints of modules is not easy, thus, it is economically advantageous to develop a rigidly connected joint for lateral-load resistance [10]. It is difficult, however, to quantitatively evaluate the structural behavior and seismic performance of the joint between the basic units of a modular system, and it is also difficult to design the joint of an existing modular system as a fully rigid joint [11]. This is because the evaluation of the linkage between units is difficult, and the relevant studies are insufficient. Thus, a connection that can serve as a moment-resisting frame needs to be suggested, and its structural performance needs to be evaluated through an experiment. For general modular design, the use of a brace or a shear wall as the lateral-load-resisting element has been investigated [12,13]. In the present study, however, securing a rigid joint performance that can induce a more efficient construction method and an integrated behavior at the joint of modular units was investigated.

### 1.2. Suggestion of a Column-Beam Joint

For a modular system, it is desirable to produce steel modular units in a factory and to finish the construction with the minimum processes on site so that the recycling of resources can be promoted and mass production can be enabled by securing a certain level of quality. Accordingly, modular units are typically connected using a connection plate and a high-strength bolt. As for the existing connection, holes are made on the members around the connection part, and high-strength bolts are fastened on the site using a hand wrench. The holes of a column, however, degrade the cross-sectional performance of the column and cause the deterioration of the on-site workability due to the use of a hand wrench. Such a joint, therefore, requires a separate lateral-force-resisting element (e.g., a brace).

**Figure 1:**
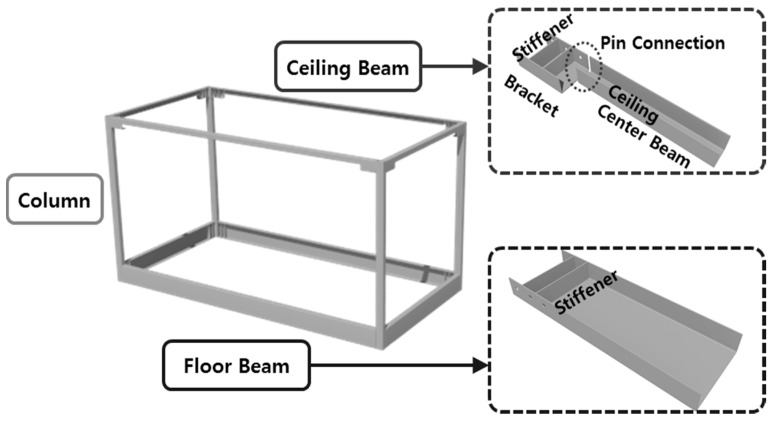
Steel frame modular unit.

**Figure 2:**
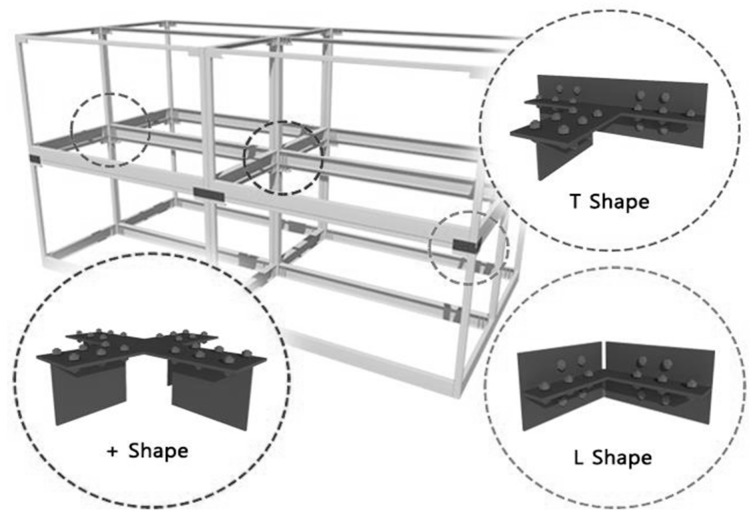
Connection attachment and the modular units.

In the present study, to make up for the aforementioned disadvantages, a basic unit was fabricated using a square-shaped steel pipe column without holes, a floor beam, central ceiling beam, and a ceiling bracket, as shown in Figure 1, and a new joint where the linkage between the basic units consists of a connection plate and a high-strength bolt was suggested, as shown in Figure 2. The suggested joint has no cross-sectional column loss and can increase the construction quality, as a high-strength bolt can be easily fastened on site. In addition, the cross-section can be optimized by using the ceiling beam for the joint between the units, and by splitting the bracket and the central beam that can increase the seismic performance. As shown in Figure 2, L-, T-, and cruciform-shaped connecting devices can be standardized depending on the module assembly. These standardized connecting devices can easily connect and disconnect basic unit modules, and can be reused.

### 1.3. Research Purpose

Steel moment-resisting frames are classified into three types: a special moment frame, an intermediate moment frame, and an ordinary moment frame. The relevant regulations reflect the results of the studies conducted based on the lessons from various earthquakes. The three steel moment frames require different levels of inelastic capability: the ordinary moment frames are close to the elastic design, whereas the special moment frames require relatively high, inelastic deformation. The seismic performance of the special moment frame is such that the earthquake energy is dissipated due to the bending collapse of the end of the beam, whereas the column and joint remain elastic. In other words, in the event of an earthquake, which is the fundamental principle of capacity design of the moment-resisting frame, the plastic hinge formation of the column is avoided in this method while the beam dissipates the input energy. This “strong column–weak beam” design concept is aimed at optimizing the energy dissipation capacity of structures, and is obtained by developing a collapse mechanism of global type [14]. For the design of the collapse mechanism of global type, the theory of plastic mechanism—based on the kinematic theorem of plastic collapse—and a design method with improved theory have been devised and developed [14,15].

When seen from the viewpoint of seismic design through energy dissipation, the standard of a joint is defined considering the P−Δ effect, depending on the plastic deformation capacity and the increase in deformation. Depending on the criterion, the joint flexural strength of a special moment frame can be defined as less than 80% of the nominal plastic flexural strength $M_{p}$ until the drift angle of 0.04 rad [16]. Therefore, in the present study, a cyclic loading test was conducted for a modular system with the suggested joint, and the seismic performance was verified. Three specimens were used, using the size and shape of the ceiling bracket as variables, since these are the most important elements for designing the suggested column–beam joint as a fully rigid connection. Additionally, one specimen for seismic-performance comparison was designed and manufactured based on the details actually applied. In order to compare the stiffness and strength of the specimen, a finite element analysis (FEA) was conducted. For FE analysis, a general-purpose finite element method (FEM) software—ANSYS (ver.10.0) [17]—was used, and the analysis was performed up to 4% drift angle.

## 2. Experiment Outline

### 2.1. Experiment Plan

**Figure 3:**
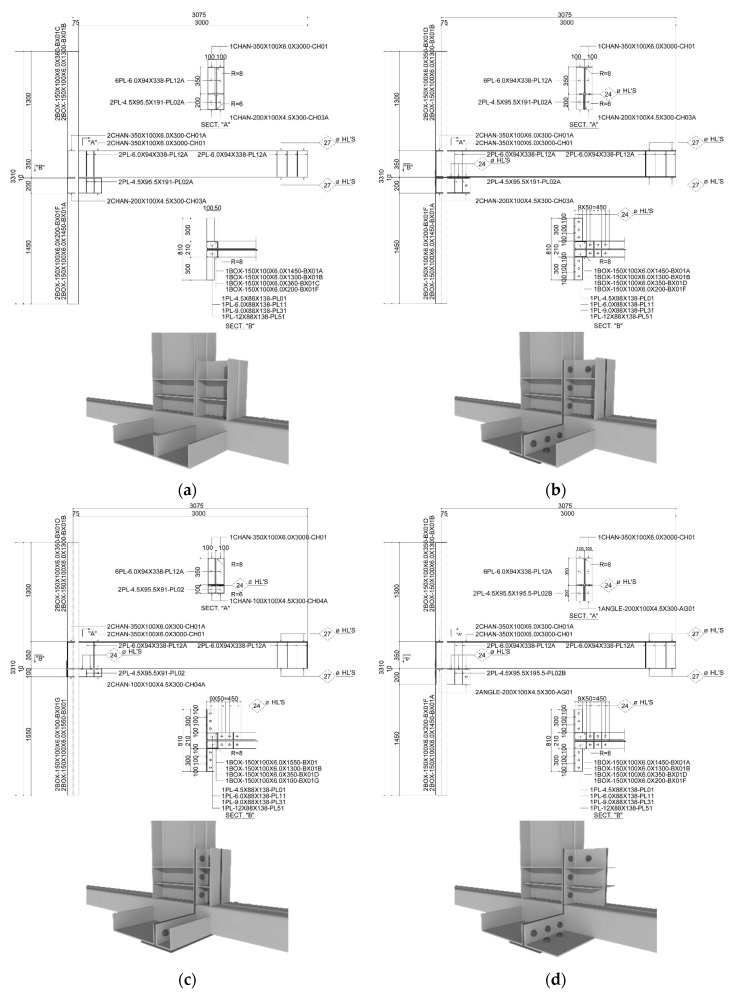
Details of the test specimens. (**a**) C200-W; (**b**) C200-B; (**c**) C100-B; (**d**) L200-B.

The specimen was an exterior joint specimen consisting of a square-shaped steel pipe column, a floor channel beam, and a ceiling bracket; the central ceiling beam was excluded because it did not contribute to the seismic performance. Table 1 summarizes the components of the specimen, depending on the variables that were used in this study. Figure 3 shows a detailed drawing of the specimen. For the column of the specimen, a square-section steel tube was used, and for the floor beam, a channel was used (see Table 1). For the ceiling bracket used as the variable, channels and an L-section steel were used, depending on the size and shape (see Table 1). The channel was manufactured by bending a steel plate; for the joint of the column, the floor beam, and the ceiling bracket, the torque-shear bolted joint, which can easily secure the standard bolt tension, was applied using a power tool. A stiffener was installed at the bolted joint of the floor beam and the ceiling bracket. By installing an internal diaphragm, the column was designed so that failure would not occur at the joint until the specimen exhibits the maximum resisting force. For the comparison specimen, C200-W, a welded joint (full-penetration welding) was applied between the column and the channels that were used as the floor beam and the ceiling beam. The initial rigidity and hysteresis characteristics of the comparison specimen were then compared with those of the remaining specimens.

The modular system forms a structure by combining units. The columns and beams of the modular building are formed by combining several units of columns and beams. The inside columns of the modular building are combined with the four-edge columns of the unit to form one column, while in the case of the inside beams, two floor beams and two ceiling brackets are combined to form one beam. From this perspective, the section property for each member of the modular building can be obtained with simple summation of each section property of the unit. The member design should satisfy the performance requirements of the element of the unit. The unit used in the specimen of this paper consisted of elements endowed with the property of slenderness, as shown in Table 2. In the table, λ represents the slenderness ratio of the column under compression, and $\lambda_{f}$ and $\lambda_{w}$ each represents the width-to-thickness ratio of the column and beam. The level of slenderness of each member can be judged by the limit $\lambda_{p}$ of the compact section and the limit $\lambda_{r}$ of the non-compact section [16]. Considering the steel used in this study, the beam flange belongs to a relatively narrow non-compact section. The fully plastic moments (Mpinc and Mpinb) of the inside column and the inside beam of the modular building obtained by simple summation are 220.02 and 216.1 kN·m, respectively. These results suggest that the inside beam of the modular building is relatively slender and weakly designed.

The distance from the loading point to the center of the column was 2875 mm. For the column, a pin was applied to both supporting points, and the distance between the supporting points was 4500 mm. The four bolts were used to connect the end of the actuator to the end of the beam, which is the loading point. The six bolts were also used to connect the column to the base block, so that distraction and slip would not occur at the loading point and the supporting point of the column until the specimen reached the maximum resistance.

### 2.2. Cyclic Loading

**Figure 4:**
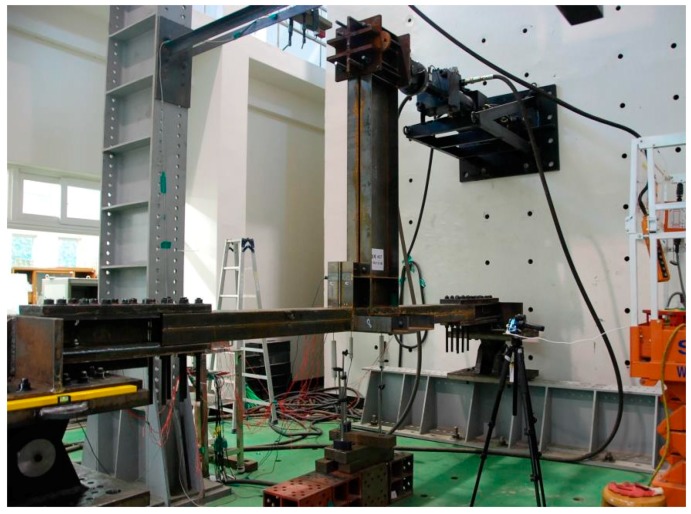
Test setup.

For the loading device, pin support was applied to both ends of the column, and an actuator was connected to the end of the floor beam, as shown in Figure 4. The experiment was conducted by laying the modular specimen that was originally in a vertical position (rotated by 90°) for the convenience of the experiment.

**Figure 5:**
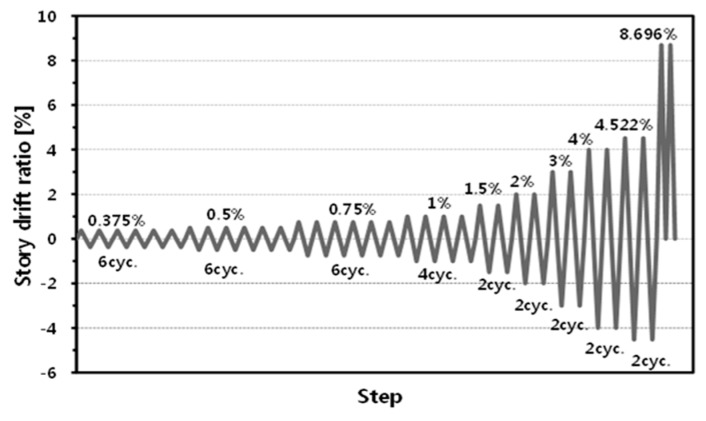
Cyclic loading pattern. cyc.: cycle.

For the loading plan, the displacement was controlled based on the loading hysteresis for the seismic-performance verification of a steel structure column–beam joint specified in KBC 2009 [16]. As for the sign of the load and displacement of the joint, the directions towards which the actuator would push and full were set to be positive (+) and negative (−), respectively. Figure 5 and Table 3 show the drift ratio and the loading cycle pattern. In the table, the 1st through 9th cycles represent the force plan that was designed to compare the results up to the 4% drift angle, which is the reference of the special moment frame connection. In the 10th cycle, the force plan was designed to be unilateral in order to analyze the end status of the structure, regardless of standards.

## 3. Experiment Results

### 3.1. Mechanical Characteristics of the Material

For the tensile test of SPSR400-grade and SPHC-grade steel [19,20] used in the experiment, three tensile specimens were manufactured, following the Test Pieces for the Tensile Test for Metallic Materials (KS B 0801) [21]. The tensile test was conducted following the Method of Tensile Test for Metallic Materials (KS B 0802) [22], and the results of the test are summarized in Table 4.

### 3.2. Theoretical and Experimental Initial Stiffness of the Specimens

**Figure 6:**
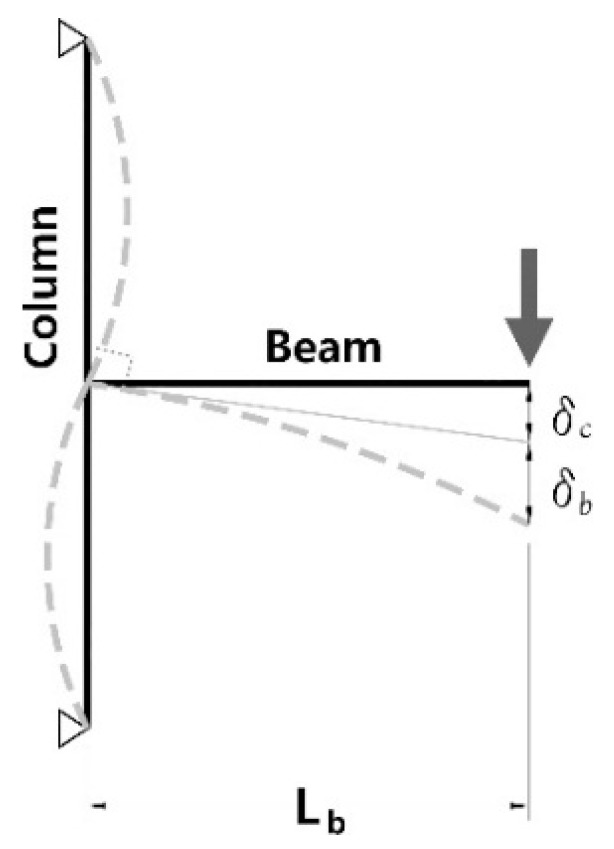
Theoretical model for fully restrained moment connection; initial stiffness of a fully restrained moment connection: Kia=P⋅Lb2δc+δb.

**Figure 7:**
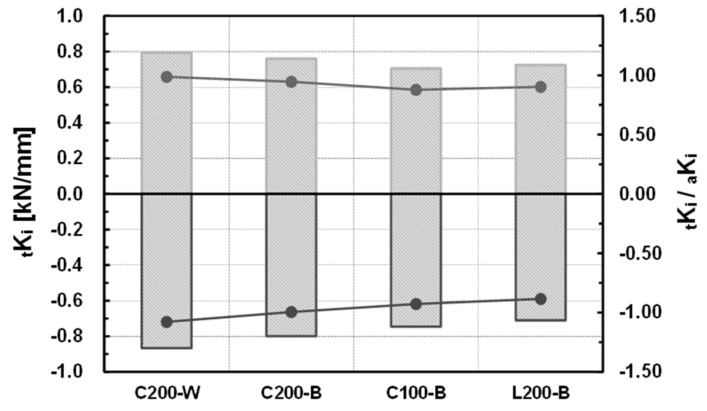
Initial stiffness of the specimens (experimental results).

The initial stiffness of the specimens was compared with the theoretical stiffness based on the mechanical model shown in Figure 6, while Table 5 and Figure 7 show the evaluation results.

The stiffness of the specimens for the (+) direction showed a 0.987–0.878 distribution relative to the initial stiffness of the specimen based on the theoretical equation. The average was 0.928, and the standard deviation was 0.048. The stiffness for the (−) direction showed a 1.079–0.884 distribution relative to the theoretical initial stiffness. The average was 0.972, and the standard deviation was 0.085. Accordingly, the initial stiffness obtained from the experiment could predict the theoretical initial rigidity based on the mechanical model, and the result was close to a rigidly connected joint.

### 3.3. Maximum Resisting Force of Each Specimen

**Figure 8:**
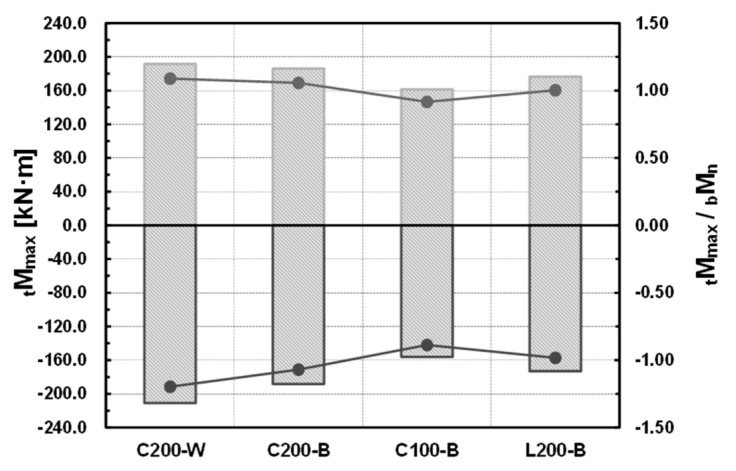
Maximum moment of the specimens (experimental results).

Using the tensile test results of the materials, the maximum resisting forces of the specimens were compared with the nominal moment strength of the floor beam based on the theoretical equation, similar to the initial stiffness examined in the previous section. The results of the comparison are shown in Table 6 and Figure 8. In this regard, the nominal moment strength, which is the theoretical maximum resisting force of the beam, is determined by the flange local buckling strength, whose value is smaller than those of the plastic moment and lateral buckling strength.

The maximum resisting forces of all the connections, excluding the (−) direction of specimens C100-B and L200-B, exceeded the theoretical nominal moment of the beam. For a total of four specimens, the maximum moment of the specimen for the (+) direction obtained from the experiment showed a 1.088–0.917 distribution relative to the theoretical nominal moment of the beam. The average was 1.016, and the standard deviation was 0.075. The maximum moment of the specimen for the (−) direction obtained from the experiment showed a 1.197–0.887 distribution relative to the theoretical nominal moment of the beam. The average was 1.034, and the standard deviation was 0.132. Accordingly, it was found that the maximum resisting force obtained from the experiment exceeded the maximum resisting force based on the theoretical equation.

### 3.4. Finite Element (FE) Modeling and Analysis Results of Each Specimen

**Figure 9:**
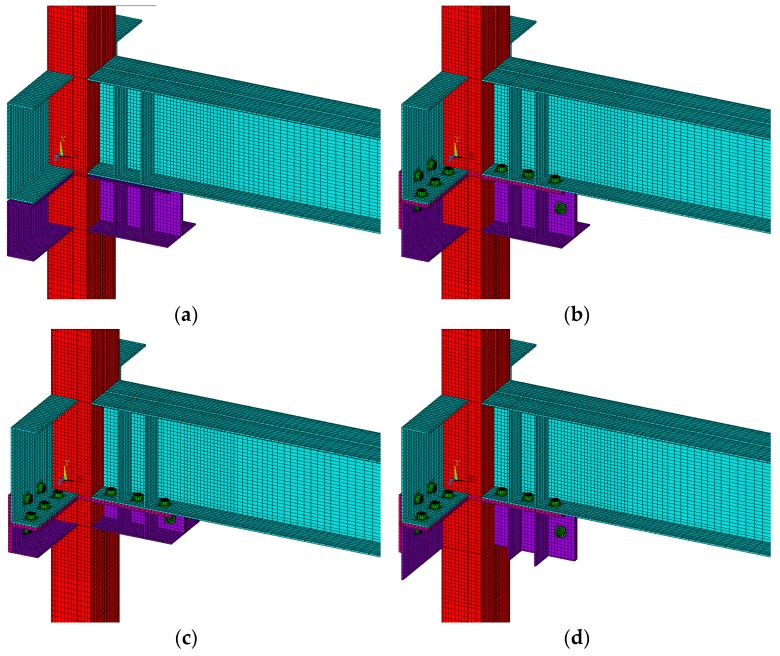
Finite element modeling of the specimens. (**a**) C200-W; (**b**) C200-B; (**c**) C100-B; (**d**) L200-B.

In order to compare the initial stiffness and strength of the specimen, ANSYS (ver.10.0) [17], a general-purpose finite element method (FEM) software, was used to perform the analysis. The adopted FEM program has the advantage of easily introducing bolt tension in the analysis of joint and convenient modeling of contact state. The finite element used in the analysis was SOLID45 and the welded parts of the modeling were not considered. In the case of the 3D solid bolt model [23], the screw part was not considered but the initial tension and contact elements of the bolt was introduced in this work. The bolt was given a design bolt (F10T-M20) tension value of 164.93 kN using PREST179 element [17]. For the interface between the steel plate and the bolt, CONTA174 and TARGE170 elements were used, respectively, with the slip coefficient assumed to be 0.5. For convenience, it is assumed that no slip occurs on the side where the bolt and washer meet, with the results of finite element modeling of the specimen shown in Figure 9.

**Figure 10:**
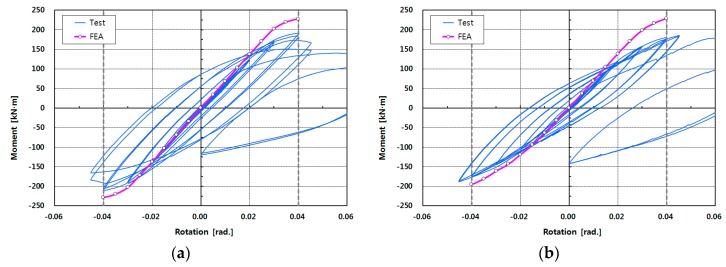

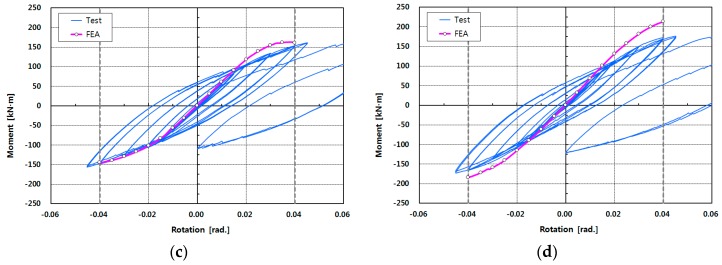
Results of finite element (FE) analysis of specimens: moment–rotation curves. (**a**) C200-W; (**b**) C200-B; (**c**) C100-B; (**d**) L200-B.

A 20 mm rigid element was modeled at the end of the column to assume both ends of the column as a hinge in the finite element modeling, before the boundary condition was applied. FE analysis should be performed according to the cyclic loading plan, but only incremental FE analysis was performed up to 4% drift angle (0.04 rad) in the (+) and (−) directions, respectively, for convenience and reduced analysis time. The results are shown in Figure 10, and the moment–rotation curve shows an inelastic behavior beyond the elastic range as the drift angle increases. As shown in the figure, the nonlinear curve of the interpretation result shows a pattern similar to the hysteretic behavior, only the larger the drift angle is, the larger the difference is.

First, the initial stiffness through the FEA and the initial stiffness of the experimental results are compared in Table 7 in order to compare the stiffness of the elastic region. The stiffness of the experimental results showed a 0.88–1.084 distribution relative to the initial stiffness of the specimen based on the FE analysis. The results are more consistent than the theoretical calculation. In other words, the initial stiffness of the FE model is more accurate than the simple initialized stiffness, and the initial stiffness of the test specimen is underestimated when reviewed based on the results of the analysis.

The results of incremental FE analysis were compared according to the drift angle, with the results shown in Table 8. This table shows the results of FE analysis and comparison for the drift angles of 0.01, 0.02, 0.03, and 0.04 rad, respectively. In the table, the moment Mθt of the specimen C200-W obtained from the experiment showed a 0.82–1.028 distribution relative to the moment MθFEA based on the FE analysis. For C200-B, C100-B, and L200-B, the moments showed 0.782–1.022, 0.865–1.084, and 0.801–0.935, respectively. The averages of the comparison results for C200-W, C200-B, C100-B, and L200-B were 0.922, 0.879, 0.961, and 0.866, respectively. Although it is relatively higher than the hysteresis curve, it shows a similar change, as described in Figure 10. Since the FE analysis is an incremental analysis, it is difficult to directly compare the results of the experiment with the cyclic load, but the similarity between the initial stiffness and the nonlinear behavior can be observed. The difference in stiffness between the test specimens of the joints also was comparatively close to the experimental results.

## 4. Comparison and Evaluation of the Test Results

### 4.1. Comparison of the Welded and Bolted Joints

**Figure 11:**
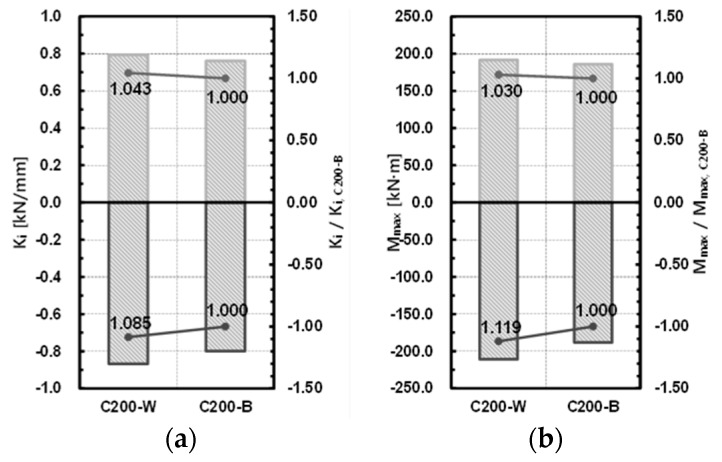
Comparison of a welded joint with a bolted joint. (**a**) Initial stiffness; (**b**) maximum strength.

For the comparison of the welded and bolted joints, specimens C200-W and C200-B were compared. The initial stiffness of the welded joint increased by about 4.3% in the (+) direction and by about 8.5% in the (−) direction compared to that of the bolted joint, and the maximum resisting force increased by about 3.0% in the (+) direction and by about 11.9% in the (−) direction. As the welded joint could secure integrity, it showed higher initial rigidity and maximum resisting force than the bolted joint. The difference from the bolted joint, which is essential for completing a modular system, was not large. The results of the comparison are shown in Table 9 and Figure 11.

### 4.2. Comparison Depending on the Shape of the Ceiling Bracket

**Figure 12:**
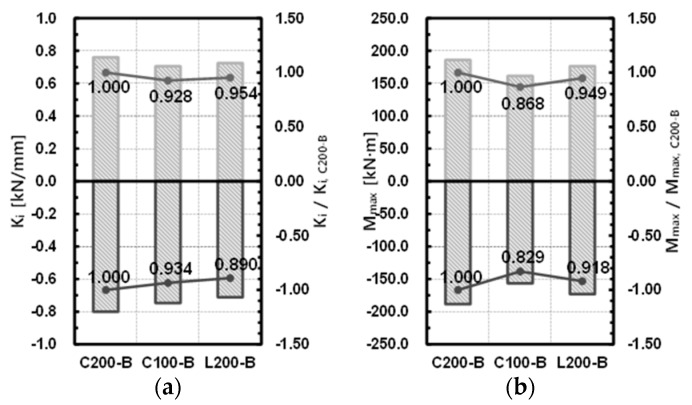
Comparison of a ceiling bracket size and shape. (**a**) Initial stiffness; (**b**) maximum strength.

For comparison, depending on the size and shape of the ceiling bracket, specimens C200-B, C100-B, and L200-B were compared. Compared to specimen C200-B, where the size of the ceiling bracket was channel 200 × 100 × 4.5 (see Table 1), the initial stiffness of specimen C100-B and specimen L200-B decreased by 7.2% and 4.6%, respectively, in the (+) direction, and by 6.6% and 11.0%, respectively, in the (−) direction; the maximum moment decreased by 13.2% and 5.1%, respectively, in the (+) direction, and by 17.1% and 8.2%, respectively, in the (−) direction. It is thought that the stiffness and resisting force of the joint decreased as the size of the ceiling bracket decreased, and the shape changed from the channel to the L-section. The results of the comparison are shown in Table 10 and Figure 12.

### 4.3. Evaluation of Seismic Performance

For the drift angle of each specimen, the rotational deformation capacity corresponding to 0.04 rad was evaluated based on a special moment frame. Considering the strength degradation occurring at the joint due to the increased deformation, the flexural strength of the joint was evaluated based on the strength for the joint of a special moment frame ($0.8M_{p}$).

**Figure 13:**
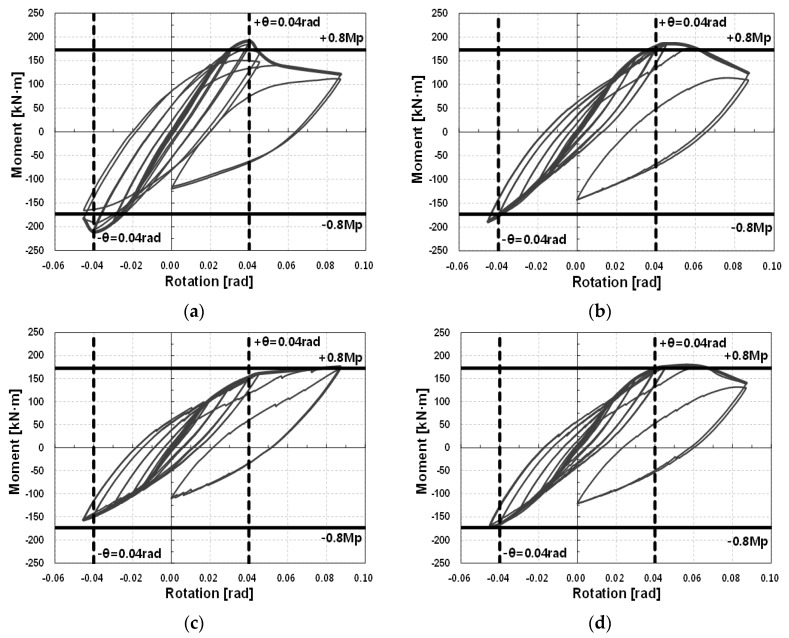
Seismic performance of the specimens: moment–rotation curves. (**a**) C200-W; (**b**) C200-B; (**c**) C100-B; (**d**) L200-B.

All four specimens satisfied the drift angle, but specimens C100-B and L200-B could not satisfy the plastic flexural strength at 0.04 rad for a special moment frame ($0.8M_{p}$). This result was predictable from the results of the previous section. In the (+) and (−) directions, specimen C200-B showed 0.04522 and 0.04525 deformation capacities, respectively, and the moments at 0.04 rad were 1.044 and 1.021, respectively, relative to the plastic flexural strength standard of a joint ($0.8M_{p}$). Thus, the specimen could satisfy the seismic-performance requirements of a special moment frame. Therefore, it is thought that when a modular system building is designed using the joint details of specimen C200-B, the special moment frame among the steel frames can be used as a seismic structure system. Table 11 and Table 12 summarize the experiment results based on the seismic-design regulations, and Figure 13 shows the seismic performance of each specimen.

## 5. Conclusions

In this study, a modular system that has no cross-sectional column loss, can be easily installed and disassembled using a power tool, and can implement a fully rigidly connected joint between units was investigated, and the seismic performance verified. To verify the seismic performance of the proposed modular system, which can enable the construction of mid- to high-rise modular buildings, a cyclic loading test was conducted using a column–beam joint system connected by a simple and standardized connecting device, and the following results were obtained.

The theoretical and experimental results were compared and analyzed with respect to the initial stiffness and the maximum resisting force. Results showed that the initial stiffness obtained from the experiment accurately predicted the theoretical value and FE analysis, and that the maximum resisting force obtained from the experiment exceeded the theoretical value. This indicates that a rigidly connected joint structural performance could be secured. The stiffness and resisting force of the modular joint decreased as the size of the ceiling bracket decreased, and the shape changed from the channel to the L-section. Accordingly, a strong column–weak beam concept can be applied in the structural design of the proposed modular building system combined in a basic unit. Based on the evaluation of the seismic performance, depending on the shape of the ceiling bracket, it is thought that when the ceiling bracket is larger than the one used for specimen C200-B, the modular system can be used as a seismic-force-resisting system to serve as a special moment frame.

In the future, an empirical evaluation needs to be performed by reflecting the experiment results for the modular system consisting of the aforementioned connection. Various studies also need to be performed using the joint method and scale as the variables.

## Figures and Tables

**Table 1:** Specimens for the cyclic loading test.

Specimen ^1^	Floor Beam ^2^	Ceiling Beam ^2^	Welding or Bolt
C200-W	Channel 350 × 100 × 6.0	Channel 200 × 100 × 6.0	Welding
C200-B	Channel 350 × 100 × 6.0	Channel 200 × 100 × 4.5	TSB, M20 (F10T) ^3^
C100-B	Channel 350 × 100 × 6.0	Channel 100 × 100 × 4.5	TSB, M20 (F10T) ^3^
L200-B	Channel 350 × 100 × 6.0	L-shaped 200 × 100 × 4.5	TSB, M20 (F10T) ^3^

^1^ Specimen name: An-B (e.g., C200-W); where, A: Ceiling bracket shape, C or L; n: Ceiling bracket depth, 100 or 200 mm; B: Joint type W (welded) or B (bolted); ^2^ Channel n1 × n2 × n3; where, n1: channel’s depth (H), n1: channel’s width (B), n3: channel’s thickness (t), and unit is mm; ^3^ TSB is a torque shear bolt; bolt diameter is 20 mm (M20) and tensile strength $F_{u}$ is 10,000 MPa (F10T) (Korea Standard-KS D 1010) [16,18].

**Table 2:** Slenderness ratio and width-to-thickness ratio of the modular unit’s elements.

Modular Unit’s Member		$\lambda =\frac{KL}{r}$	$\lambda_{f}=\frac{H}{t}$	$\lambda_{w}=\frac{B}{t}$
Channel *H* × *B* × *t*	Floor beam	-	16.67	56.33
350 × 100 × 6.0			Non-compact	Compact
Square-section *H* × *B* × *t*	Column	69.29	13.67	22.0
150 × 100 × 6.0			Compact	Compact

*H*, *B* and *t* are the depth, width and thickness of channel, respectively and unit is mm. K, L, and r are the effective length factor of column, unsupported length of column, and the radius of gyration of column’s section, respectively.

**Table 3:** Loading plan for the cyclic loading test.

Step	Drift Ratio (%)	Displacement (mm)	Cycle	Rate (mm/s)
1	0.375	10.781	6	0.250
2	0.500	14.375	6	0.250
3	0.750	21.563	6	0.250
4	1.000	28.750	4	0.333
5	1.500	43.125	2	0.500
6	2.000	57.500	2	1.000
7	3.000	86.250	2	1.000
8	4.000	115.000	2	1.000
9	4.522	130.000	2	1.000
10	8.696	250.000	2 (only (+) direction)	1.000

**Table 4:** Results of a tensile test.

Plate Name	Yield Strength (MPa)	Tensile Strength (MPa)	Yield Ratio (%)	Elongation (%)	Site
PL-6 (SPSR400)	401.5	464.2	86.5	16.5	Column
PL-6 (SPHC)	299.2	369.8	80.9	30.6	Floor beam
PL-4.5 (SPHC)	248.1	383.0	64.8	30.6	Ceiling Bracket

**Table 5:** Initial stiffness of the test specimens.

Specimen.	Kit (kN/mm)		KitKia	
	(+) Direction	(−) Direction	(+) Direction	(−) Direction
C200-W	0.793	0.867	0.987	1.079
C200-B	0.760	0.799	0.946	0.995
C100-B	0.705	0.746	0.878	0.928
L200-B	0.725	0.711	0.902	0.884

Kit: Initial stiffness (experiment); Kia: Initial stiffness (theory).

**Table 6:** Maximum strength of the test specimens.

Specimen	Mmaxt (kN·m)		MmaxtMnb	
	(+) Direction	(−) Direction	(+) Direction	(−) Direction
C200-W	191.57	210.78	1.088	1.197
C200-B	185.99	188.37	1.056	1.070
C100-B	161.39	156.22	0.917	0.887
L200-B	176.43	173.00	1.002	0.983

Mmaxt: Maximum moment of the specimens (experiment); Mnb: Nominal flexural strength (flange local buckling).

**Table 7:** Initial stiffness of the FE model (FEA).

Specimen	KiFEA (kN/mm)		KitKiFEA	
	(+) Direction	(−) Direction	(+) Direction	(−) Direction
C200-W	0.830	0.831	0.955	1.043
C200-B	0.845	0.769	0.899	1.039
C100-B	0.742	0.688	0.951	1.084
L200-B	0.824	0.743	0.880	0.957

Kit: Initial stiffness (experiment); KiFEA: Initial stiffness (FEA).

**Table 8:** Moment of the FE model (FEA).

Specimen		MθFEA (kN·m)				MθtMθFEA			
		0.01 rad	0.02 rad	0.03 rad	0.04 rad	0.01 rad	0.02 rad	0.03 rad	0.04 rad
C200-W	(+) Dir.	68.623	137.328	202.687	228.052	0.931	0.896	0.841	0.820
	(−) Dir.	68.683	137.542	202.899	228.579	1.028	1.016	0.934	0.910
C200-B	(+) Dir.	69.860	138.728	198.231	228.633	0.884	0.867	0.795	0.782
	(−) Dir.	63.581	119.119	160.871	195.380	1.022	0.885	0.898	0.900
C100-B	(+) Dir.	61.295	117.714	154.436	161.109	0.925	0.892	0.865	0.950
	(−) Dir.	56.857	102.196	129.630	144.844	1.084	0.978	0.980	1.017
L200-B	(+) Dir.	68.077	131.277	181.565	212.987	0.873	0.860	0.826	0.801
	(−) Dir.	61.402	115.227	158.872	184.281	0.935	0.882	0.858	0.896

Mθt: Moment of the specimens (experiment); MθFEA: Moment of the FE models (FEA). Dir.: Direction.

**Table 9:** Comparison of a welded joint with a bolted joint.

Specimen	Initial Stiffness				Maximum Strength			
	Kit (kN/mm)		KitKtargett		Mmaxt (kN·m)		MmaxtMtargett	
	(+) Dir.	(−) Dir.	(+) Dir.	(−) Dir.	(+) Dir.	(−) Dir.	(+) Dir.	(−) Dir.
C200-W	0.793	0.867	1.043	1.085	191.6	210.8	1.030	1.119
C200-B	0.760	0.799	1.000	1.000	186.0	188.4	1.000	1.000

Ktargett: Kit, of the specimen C200-B; Mtargett: Mmaxt of the specimen C200-B. Dir.: Direction.

**Table 10:** Comparison of a ceiling bracket size and a shape.

Specimen	Initial Stiffness				Maximum Strength			
	Kit (kN/mm)		KitKtargett		Mmaxt (kN·m)		MmaxtMtargett	
	(+) Dir.	(−) Dir.	(+) Dir.	(−) Dir.	(+) Dir.	(−) Dir.	(+) Dir.	(−) Dir.
C200-W	0.705	0.746	0.928	0.934	161.4	156.2	0.868	0.829
C200-B	0.725	0.711	0.954	0.890	176.4	173.0	0.949	0.918

Ktargett: Kit, of the specimen C200-B; Mtargett: Mmaxt of the specimen C200-B.

**Table 11:** Comparison of a moment for a special moment frame.

Specimen	(+) Direction		(−) Direction	
	M+t (kN·m)	M+t0.8Mp	M−t (kN·m)	M−t0.8Mp
C200-W	191.57	1.108	210.60	1.218
C200-B	180.55	1.044	176.57	1.021
C100-B	153.80	0.890	148.40	0.858
L200-B	171.50	0.992	165.82	0.959

**Table 12:** Comparison of a drift angle for a special moment frame.

Specimen	(+) Direction		(−) Direction	
	θmax+t (rad)	θmax+t0.04	θmax−t (rad)	θmax−t0.04
C200-W	0.04526	1.132	0.04516	1.129
C200-B	0.04522	1.130	0.04525	1.131
C100-B	0.04517	1.129	0.04525	1.131
L200-B	0.04524	1.131	0.04524	1.131

## References

[1] Jellen A., Memari A. The State-of-the-Art Application of Modular Construction to Multi-Story Residential Building. Proceedings of the 1st Residential Building Design & Construction Conference 2013.

[2] Kim J., Lee J. (2014). A basic study on the application of modular construction. J. Korean Hous. Assoc..

[3] Lu N., Korman T. Implementation of Building Information Modeling (BIM) in Modular Construction: Benefits and Challenges, Construction Research Congress; Innovation for Reshaping Construction Practice. Proceedings of the 2010 Construction Research Congress.

[4] Kim G., Lee J., Son S., Lee B. (2014). An experimental study on 2 hours fire resistance performance of load bearing walls used in modular buildings. J. Arch. Inst. Korea Struct. Constr..

[5] Annan C.D., Youssef M.A., El Naggar M.H. (2009). Experimental evaluation of the seismic performance of modular steel-braced frames. Eng. Struct..

[6] Olearczyk J., Al-Hussein M., Bouferguene A. (2014). Evolution of the crane selection and on-site utilization process for modular construction multilifts. Autom. Constr..

[7] Hong S., Cho B., Chung K., Moon J. (2011). Behavior of framed modular building system with double skin steel panels. J. Constr. Steel Res..

[8] Lawson R., Ogden R., Bergin R. (2012). Application of Modular Construction in High-Rise Buildings. J. Archit. Eng. ASCE.

[9] Shon S., Kwak E., Lee S. (2015). A Study of Modular Dome Structural Modeling with Highly Filled Extrusion Wood-Plastic Composite Member. J. Korea Inst. Struct. Maint. Insp..

[10] Ha T., Cho B., Kim T., Lee D., Eom T. (2013). Earthquake resistance of modular building units using load-bearing steel stud panels. J. Korean Soc. Steel Constr..

[11] Choi K., Lee H., Kim H. (2016). Influence of Analytical Models on the Seismic Response of Modular Structures. J. Korea Inst. Struct. Maint. Insp..

[12] Kim T., Wilcoski J., Foutch D.A., Lee M. (2006). Shaketable tests of a cold-formed steel shear panel. Eng. Struct..

[13] Moghimi H., Ronagh H.R. (2009). Better connection details for strap-braced CFS stud walls in seismic regions. Thin-Walled Struct..

[14] Montuori R., Nastri E., Piluso V. (2015). Advances in theory of plastic mechanism control: Closed form solution for MR-Frames. Earthq. Eng. Struct. Dyn..

[15] Montuori R., Muscati R. (2016). A general design procedure for failure mechanism control of reinforced concrete frames. Eng. Struct..

[16] Architectural Institute of Korea (2009). Korean Building Code and Commentary-Structural.

[17] Kohnke P., ANSYS (1999). Theory Reference—Release 5.6.

[18] Korean Agency for Technology and Standards, KS D 1010 (2014). Set of High Strength Hexagon Bolt, Hexagon Nut and Plain Washers for Friction Grip Joints.

[19] Korean Agency for Technology and Standards, KS D 3501 (2013). Hot-Rolled Mild Steel Plates, Sheets and Strip.

[20] Korean Agency for Technology and Standards, KS D 3568 (2014). Carbon Steel Square Pipes for General Structural Purposes.

[21] Korean Agency for Technology and Standards, KS B 0801 (2007). Test Pieces for Tensile Test for Metallic Materials.

[22] Korean Agency for Technology and Standards, KS B 0802 (2013). Method of Tensile Test for Metallic Materials.

[23] Kim J., Yoon J., Kang B. (2007). Finite element analysis and modeling of structure with bolted joints. Appl. Math. Model..

